# Short- and Long-Term, 11–22 Years, Results after Laparoscopic Nissen Fundoplication in Obese versus Nonobese Patients

**DOI:** 10.1155/2017/7589408

**Published:** 2017-05-11

**Authors:** Mario Schietroma, Federica Piccione, Marco Clementi, Emanuela Marina Cecilia, Federico Sista, Beatrice Pessia, Francesco Carlei, Stefano Guadagni, Gianfranco Amicucci

**Affiliations:** Department of Surgery, University of L'Aquila, L'Aquila, Italy

## Abstract

**Background:**

Some studies suggest that obesity is associated with a poor outcome after Laparoscopic Nissen Fundoplication (LNF), whereas others have not replicated these findings. The effect of body mass index (BMI) on the short- and long-term results of LNF is investigated.

**Methods:**

Inclusion criteria were only patients who undergone a LNF with at least 11-year follow-up data available, patients with preoperative weight and height data available for calculation of BMI (Kg/m^2^), and patients with a BMI up to a maximum of 34.9.

**Results:**

201 patients met the inclusion criteria: 43 (21.4%) had a normal BMI, 89 (44.2%) were overweight, and 69 (34.4%) were obese. The operation was significantly longer in obese patients; the use of drains and graft was less in the normal BMI group (*p* < 0.0001). The hospital stay, conversion (6,4%), and intraoperative and early postoperative complications were not influenced by BMI.

**Conclusions:**

BMI does not influence short-term outcomes following LNF, but long-term control of reflux in obese patients is worse than in normal weight subjects.

## 1. Introduction

Gastroesophageal reflux disease (GERD), recognized as a clinical entity only in the mid-1930s, is now the most common upper gastrointestinal disease in the Western Countries, with 10%–20% of the population experiencing weekly symptoms [1]. Its prevalence is also increasing in the Far East (Japan) and other areas in Asia [2]. This may be related to increased fat consumption in the diet, and the expanding proportion of obese individuals [3]. In fact obesity has long been known to be a risk factor for the development of GERD.

The availability of laparoscopic antireflux surgery (LARS) has changed the threshold for referring patients to surgery. Several studies have reported excellent short- [4] and long-term [5, 6] results for this procedure.

A number of studies have investigated the relationship between obesity and outcome following laparoscopic antireflux surgery [7–17], although the data from these studies have been confusing, with some studies suggesting that obesity is associated with a poorer outcome [7, 8, 12], whereas others have not replicated these findings [9–11, 13–17]. Recently, Telem et al. [18], in a retrospective review of 4.231 obese and morbidly obese patients who underwent fundoplication for GERD, have demonstrated that a laparoscopic antireflux surgery can be performed in the setting of obesity with no difference in the overall or individual postoperative complication or mortality. However, as the authors have clearly stated, “the durability of this operation remains unknown. Functional outcomes following fundoplication in our obese patient cohort were not able to be assessed and remain a source of debate within the literature.” Therefore, in this study, the long-term efficacy (for at least 11 years) of Laparoscopic Nissen Fundoplication (LNF) in controlling reflux with respect to BMI was investigated in a prospective fashion. Furthermore we examined the effect of preoperative body mass index (BMI) on the immediate operative outcome and complications of LNF.

## 2. Methods and Materials

**Figure 1:**
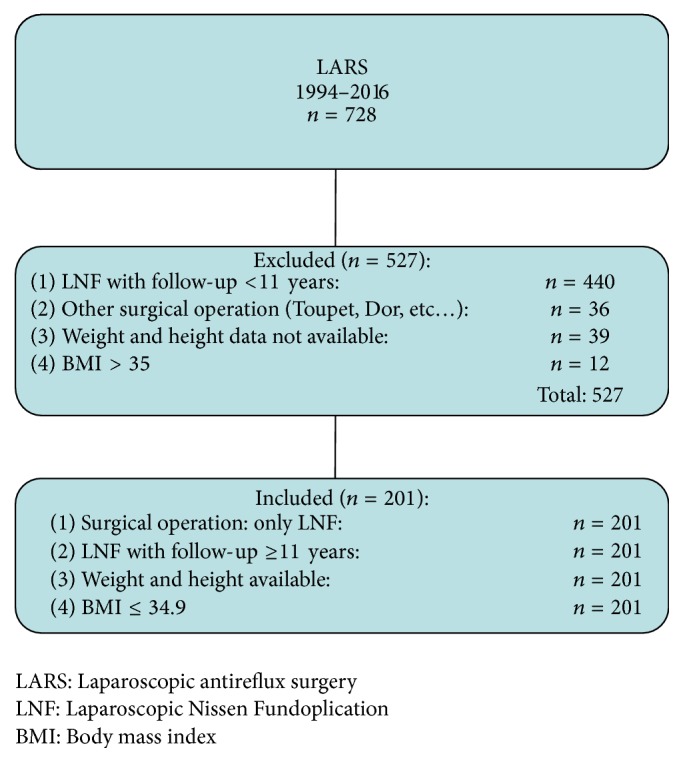
Inclusion and exclusion criteria.

This study was a retrospective analysis of prospectively collected data. Between April 1994 and October 2016, 728 consecutive patients underwent LARS for symptomatic gastroesophageal reflux disease. In this study we included patients that met the following criteria (Figure 1): LNF with at least 11-year follow-up data available (April 1994–April 2004) and preoperative weight and height data available for calculation of body mass index (Kg/m^2^). Patients were divided into three BMI groups according to the WHO classification: normal weight (BMI < 25), overweight (BMI 25–29.9), and obese (BMI > 30). The outcome was determined for each category of BMI. Patients with BMI > 35 with obesity-related comorbidities were candidates for morbid obesity surgery instead of laparoscopic antireflux surgery procedure and therefore were not included in the study (Figure 1). All patients referred to us with probable GERD (esophageal symptoms and/or extraesophageal symptoms) were comprehensively evaluated. The study protocol was approved by the Ethical Committee of the Faculty of Medicine of the University of L'Aquila.

### 2.1. Preoperative Studies

Performed preoperatively laboratory investigations included upper alimentary endoscopy [19], esophagogram, stationary esophageal high-resolution manometry [20], ambulatory 24-hour pH-impedance testing of the esophagus [21–24], and upper abdominal ultrasound.

### 2.2. Indications for Surgery

Indications for surgery were failed medical management (inadequate symptom control, or medication side effects); patients who opt for surgery despite successful medical management (due to quality of life considerations, life-long need for medication intake); complications of GERD (Barrett's esophagus, peptic stricture); and extraesophageal manifestations (asthma, hoarseness, cough, chest pain, and aspiration).

### 2.3. Operative Technique

All operations were performed by a single surgeon. Modified LNF was performed by fashioning a floppy 360° posterior wrap with circumferential dissection and mobilization of the esophagus without routine division of the short gastric vessels. Posterior hiatal repair was not routinely performed but only in patients with hiatus hernia. In the last case mentioned, graft usage was found to be more preferable. If hiatal hernia was ≤3 cm, posterior hiatal repair was performed by positioning of not resorbable mesh (PolyTetraFluoroEthylene, PTFE). Dimensions of mesh used vary from 2 × 4 cm to 5 × 6 cm, profiled to “U” form; if hiatal hernia was >3 cm, posterior hiatal repair was performed by positioning of mesh and stiches. No intraesophageal bougie was used during the creation of the wrap. A 1,5–2 cm wrap (short) was created with the naked eye with two or three nonabsorbable suture (floppy) and the anterior esophageal wall was not included. Both vagal trunci were identified and included in the wrap. At the end of the operation, the looseness of the wrap is confirmed by passing a blunt laparoscopic instrument between the wrap and the distal aspect of the esophagus. The use of drains in patients was based on the surgeon's discretion. Obese patients received routine prophylaxis with subcutaneous low-molecular-weight heparin during the induction of anesthesia, in addition to compression stockings.

### 2.4. Postoperative Care

All patients were evaluated 1 week and 3 months after surgery and yearly thereafter. Patients who could not come for their yearly visit were contacted by phone and asked about their symptom status. In all patients (symptomatic and asymptomatic) gastroscopy and pH metry were performed. These studies were performed at presentation of symptoms in symptomatic patients and every year in asymptomatic patients.

The database, used to collect information, included the following details: patient age at the moment of operation, type of fundoplication performed, duration of the operation, eventual conversion from laparoscopic to an open procedure, intra- and early postoperative complications, late outcomes, timing, and reasons for any revisional surgery. Complication severity was graded according to the Dindo-Clavien classification [25].

All patients included in the study had been followed up for at least 11 years.

An intention-to-treat analysis was performed. Patients who required conversion to open procedure, as well as those requiring later surgical revision, were included in the analysis.

### 2.5. Statistical Analysis

Statistical analysis was performed using commercially available statistical software (GraphPad InStat, version 3.06 for Windows Vista, GraphPad Software, San Diego California USA, http://www.graphpad.com/). Spearman rank correlation, ANOVA, and chi-squared tests were used to determine the significance of any differences between the study groups. Statistical significance was determined if *p* values were less than 0.05.

## 3. Results

### 3.1. Preoperative Assessment

Out of 728 patients, 201 met the inclusion criteria for this study (Figure 1).  Table 1 reported baseline characteristics of patients. There were no statistically significant differences between normal BMI, overweight, and obese patients in terms of age, gender (Table 1), type and duration of symptoms, endoscopic, and manometric and pH-metric data (Table 2). Moreover there were no differences in ASA scores among the BMI group. Hiatal hernia was encountered less frequently in the normal BMI group and this difference was statistically significant (*p* < 0.01) (Table 2). Mean follow-up was 16.5 years (range 11–22 years) (Table 1). Forty-three (21.4%) patients had a normal BMI, 89 (44.2%) were overweight, and 69 (34.3%) were obese (Table 1).

### 3.2. Operative and Postoperative Outcome

The duration of the operation was significantly longer in obese patients, and the use of drains and grafts for hiatal hernia repair was less in the normal BMI group (*p* < 0.0001) (Table 3). The higher number of grafts used in higher-BMI patients can be explained by the significantly higher number of hiatal hernias in overweight and obese patients. The hospital stay did not differ among the groups.

#### 3.2.1. Conversion

Thirteen (6.4%) patients required conversion from a laparoscopic to an open procedure, nine out of the first 50 cases of the series, two among cases 51–100, and two among cases 100–201. Two (4.6%) of these were of normal weight, six (6.7%) were overweight, and five (7.2%) were obese (Table 1). Conversion to an open surgical operation was not influenced by preoperative weight. The following conditions required conversion to an open laparotomy: inability to reduce a very large hiatal hernia (6 patients: 1 normal weight, 3 overweight, and 2 obese), dense upper abdominal adhesions (5 patients: 3 overweight, and 2 obese), and technical difficulties with esophageal dissection due to periesophagitis (2 patient: 1 normal weight, and 1 obese). During the 16-year mean follow-up, pH metry-proven reflux recurrence occurred in 2 patients (obese group). Insufficiency of the fundoplication (wrap undone) was diagnosed in one patient who subsequently underwent a laparotomic reoperation after 13 years.

#### 3.2.2. Complications

Twelve intraoperative complications in 12 patients and 23 postoperative complications in 21 patients occurred. Most of postoperative complications were minor (Clavien 1-2; *n* = 19), while major complications (Clavien 3-4) were four: pneumonia occurred in three cases and was treated successfully with antibiotics. One intra-abdominal abscess was diagnosed 9 days after the operation and treated conservatively without drainage of grade 5 occurring (absence of mortality).

The three groups were similar regarding the rate of intraoperative and early postoperative complications.

### 3.3. Long-Term Follow-Up Data (Tables 4 and 5)

A total of 34 patients (16.9%) reported dysphagia 2 months after the operation, but these symptoms persisted in only 7 patients (3.4%) at 6 months. Out of these 7 patients, 3 presented with severe dysphagia. Five were in the first 50 cases of operative series. Dysphagia, resolving spontaneously or requiring reintervention (dilatation or reoperation), was distributed evenly among the groups. Four patients (2%) required endoscopic dilatation. Three patients were successfully managed with a single dilatation procedure, while one patient required several dilatations before the condition of suitable swallowing was achieved. Three patients (1.5%) required reoperation for prolonged dysphagia (2 for a tight wrap and 1 for a tight esophageal hiatus) after failed dilatation attempts. All underwent laparoscopic conversion from Nissen procedure to Toupet, with enlargement of hiatal opening in one. Dysphagia was resolved completely in all patients. None of the patients who required dilatation or reoperation had preoperative endoscopic evidence of an esophageal stricture, whereas 1 patient reported no improvement of dysphagia postoperatively (Table 4).

The rate of bloating was evenly distributed among the groups (Table 4). During the 16-year mean follow-up, pH metry-proven reflux recurrence occurred in 27 patients, giving an overall recurrence rate of 13.4% (Table 4). All patients had pathologic acid exposure time and a positive DeMeester score (Table 5). A positive SI and SAP was present in 22 patients (81.4%) (Table 5). A striking correlation existed between recurrence rate and BMI. Of the obese patients, 27.5% had failed operations, in contrast to only 2.3% of normal and 7.8% of overweight patients. Insufficiency of the fundoplication (wrap undone) was diagnosed in four patients, who underwent reoperation, three by laparoscopy after 7 years (obese group), 9 years (overweight group), and 11 years (obese group), respectively, and one by laparotomy after 3 years (obese group). Once, a patient's symptoms were treated with medication only.

A barium contrast study showed an intrathoracic herniation of the fundoplication in 3 patients, with severe regurgitation. This failure was among patients who had a fundoplication only. These patients underwent reoperation by laparoscopy after 4, 8, and 10 years. At this writing, all patients who underwent a reoperation (3 for dysphagia, 4 for wrap undone, and 3 for fundoplication herniation) are free of symptoms.

Two incisional hernias (one overweight group and one obese group) were corrected.

Two patients with BMI > 35 who had refused bariatric surgery underwent LNF. Both these patients required conversion from laparoscopic to open procedure: in one case for the inability to reduce a very large hiatal hernia and in the other for the presence of severe periesophagitis. During the follow-up, 13 and 15 years, respectively, pH metry has proven reflux recurrence in one patient, treated with medication only. These patients were excluded from the study, since this data is not statistically significant.

## 4. Discussion

Over the last fifteen years the advent of laparoscopic surgery has changed the way in which antireflux surgery is performed, with the associated advantages of minimally invasive surgery, rendering esophageal wrapping more acceptable [6, 26].

Obesity has long been known to be a risk factor for the development of gastroesophageal reflux disease. It is also thought to be associated with an increased risk of a poorer clinical outcome following antireflux surgery, specifically due to recurrent reflux or paraesophageal hiatus herniation. However there seems to be an increasing trend to use antireflux surgery as treatment for reflux in the obese patients [17]. During the last decade a number of studies have assessed the probable adverse effects of obesity on the surgical outcome of LARS [7–17]. Interestingly enough, results of previous studies were conflicting, with some studies suggesting that obesity is associated with a poorer outcome [7, 8, 12], whereas others have not replicated these findings [9–11, 13–17].

D'Alessio et al. [9], Winslow et al. [10], and Ng et al. [15] have found that symptom relief and complications rate were similar in all BMI groups. However, these studies had a short follow-up. In the study of Chisholm et al. [16] the clinical outcomes were unaffected by BMI. In this study mean follow-up was 7.5 years (range 1–15 years), but was retrospective.

Tekin et al. [17] report a single surgeons' experience with 1,000 consecutive patients and to our knowledge it is the largest series from a single centre that addresses this issue in a prospective fashion. They affirm that “long-term control of reflux by LARS in obese patients is good but slightly worse than that in normal weight subjects regardless of the type of the operation performed. Obesity per se is not a contraindication to LARS.” In this study mean follow-up was 53.33 ± 17.21 months.

In contrast other studies have demonstrated that antireflux surgery is associated with a poorer outcome in obese patients [7, 8, 12].

Perez et al. in a retrospective study [7] found a correlation between recurrent reflux and BMI, independent of the type of fundoplication performed (BMI > 30 = 31%, BMI < 30 = 4.5%).

In this study mean follow-up was 33 months.

The only other study that reported a significantly increased recurrence of reflux after the Nissen procedure in obese subjects was also retrospective [8].

Therefore most previous aforementioned studies addressing this issue as weaknesses because of either a short follow-up [9, 10, 15] (with the exception of the study of Chisholm et al.) [16] or the use of a nonstandardized surgical approach as different surgeons from multiple centres were involved (a part from study of Tekin et al.) [17]. Furthermore, the retrospective nature of some of the studies [7, 8, 12, 16] was an additional short-coming of most of these papers.

In the present study all data were prospectively collected, all operations were performed by a single surgeon, and all patients have been followed for at least 11 years after their original operation (mean 16.5 years, range 11–22 years).

In our series, increased BMI was associated with a slight increase in age (Table 1), in duration of the symptoms, erosive nature of the disease, and Barrett's metaplasia, although the difference was not statistically different (Table 2). The rate of hiatal hernia was also higher in patients with increased BMI, and the difference was statistically different. An increased number of hiatal hernias in obese subjects was also reported by other studies [10, 11, 15, 16].

Almost all previous studies reported longer operating times for LARS in obese subjects [9–11, 14–17], and Ng et al. [15] reported a twofold higher rate of operational difficulty in assessing visual access, intra-abdominal bleeding, and pleural tear. Tekin et al. [17] also reported an increased difficulty in performing LARS in an obese subject. It is noteworthy, however, that this difficulty never resulted in conversion, major complications, or delay in discharge. The increased difficulty in performing LNF in an obese subject was also evident in our series as indicated by the longer operating times, higher rate of hiatal hernia, and graft and drain usage (Table 3). This difficulty required conversion to open laparotomy in 5 (7.2%) obese patients, whereas 2 (4.6%) patients of normal weight group and 6 (6.7%) of overweight group underwent conversion from a laparoscopic to an open procedure. However this difference and the longer time to discharge in obese patients were not significant.

All previous studies [9, 10, 14–16] but one [12] showed no significant increase in perioperative complications after LARS laparoscopic antireflux surgery in obese subjects. In our series there was not a significantly increased risk associated with LNF in higher-BMI patients with respect to operative and early postoperative complications.

Postoperative long-term problems such as dysphagia and bloating were distributed evenly among the BMI groups in the present series. Reoperation due to dysphagia was performed only in 3 cases (1.5%) and endoscopic dilatation in 4 cases (2%). Five of them were in the first 50 cases of the operative series. The high rate of troublesome dysphagia necessitating reintervention at 7 months was no longer observed at 5 and 11 years and beyond follow-up.

The rate of dilatation and reoperation for dysphagia was higher in patients with normal weight, but the difference was not statistically different (Table 4). There is no detailed information regarding the effect of BMI on such late outcome parameters after LARS in most of the previous literature. Nevertheless, all studies [9, 10, 15–17] reported no effect of BMI on general dysphagia status after LARS. Bloating was also evenly distributed in all BMI categories [10, 16, 17].

One of the most important outcome parameters, namely, the problem of recurrence, deserves special attention. Surgical expertise had been credited for achieving better recurrence rates, but reported recurrence rates of reflux after LARS differ greatly from one series to another, depending on how the recurrences were defined. In our study the pH monitoring was assessed to define recurrent reflux. Actually ambulatory pH monitoring is the most objective assessment whether or not the patient has GERD [27]. Indeed several studies have shown that an abnormal 24-h pH score is the best predictor of a successful surgical outcome [13]. Prolonged pH monitoring (48 h or more) likely increases sensitivity to detect pathological increased esophageal acid exposure [28, 29]. It should also be noted that heartburn score and PPI (Proton Pump Inhibitor) use do not provide objective evidence of recurrent gastroesophageal reflux [30]. Rather the score is a patient-reported score for the symptom of “heartburn,” which relies on how each individual interprets this symptom [30]. Other studies have shown that only 30–35 per cent of PPI use after antireflux surgery is actually for recurrent gastroesophageal reflux [31–33]. Although other studies have demonstrated that the heartburn score does correlate with reflux [34–37], Wijnhoven [30] affirms that would be desirable to validate these outcomes with pH monitoring. It is well know that aggressive follow-up protocols by means of routine postoperative pH metry and endoscopic control would result in much higher recurrence rates. Furthermore, longer follow-up periods will also result in higher recurrence rate.

In our study patients with a higher-BMI had a statistically significant increase in recurrence rates (Table 4). Both groups of patients with BMI between 25 and 30 and BMI > 30 both had significantly higher recurrence rates than that observed in normal weight subjects. It is also very important to note that the mean follow-up duration was 16.5 years (range 11–22 years) and there was no difference in the mean follow-up duration between normal and obese patients (Table 1). Moreover our aggressive follow-up protocols by routine postoperative pH metry and endoscopic control would explain higher recurrence rates as regards studies [17]. We found that while obese patients had similar short outcomes than other patients (*p* = 0.39) obese patients had a higher failure rate (*p* = 0.036) after follow-up of 11 years or more.

The precise mechanism by which obesity adversely affects the durability of antireflux operations is not clear. Antireflux operations can fail from loosening of the fundoplication, slippage of the repair, or migration of the wrap into the chest [38–42]. Fixation of the fundoplication to the undersurface of the diaphragm seems less effective in preventing this complication than thorough esophageal mobilization and crural closure [42]. The crural closures were not routinely closed in our study, but the fundoplication herniation occurred only in obese patients.

The data in our study does not provide a mechanistic reason for the failure of antireflux operations due to obesity. The esophageal hiatus is a very dynamic area, moving with each breath and each swallow. We can only theorize that increased intra-abdominal pressure in obese patients augments the usual wear and tear on the surgical repair and contributes to loosening of the crural closure and fundoplication.

Moreover a variety of mechanisms have been described that likely contribute to the association of GERD and obesity. These mechanisms include diminished lower esophageal sphincter pressure, hypertensive contractions of esophagus (“nutcracker esophagus”), disordered contractions of esophagus (nonspecific motility disorder), increased frequency of transient LES relaxations (TLESRs) [43–46], gastric motor abnormalities (gastroparesis) [47, 48], and presence of hiatal hernia [35, 41]. Presence of these alterations in obese patients should furthermore clarify not excellent results in patients in which fundoplication was performed. In our study we observed the same result. Therefore we agree with authors who affirm that for obese patients suffering from GERD weight loss in conjunction with antisecretory medications is first-line therapy [49]. Indeed, evidence supports the role of weight loss as a beneficial therapy for reflux symptoms [50, 51]. When medical efforts to lose weight fail, bariatric surgical procedure are considered (laparoscopic adjustable gastric band; vertical banded gastroplasty; Roux-en-y gastric bypass) [51, 52]. The effects of surgery on reflux symptoms are twofold in that these procedures reduce the BMI of patients and also physically alter the anatomy of the gastrointestinal tract. The outcomes of such procedures have been the focus of many studies [53–55]. The Roux-en-y gastric bypass (RYGB) has demonstrated consistently favourable results as an antireflux procedure in several studies [56–59].

On balance, these studies provide strong evidence favouring RYGB surgery as a therapy for patients with morbid obesity suffering from concomitant GERD.

## 5. Conclusions

In conclusion, we have demonstrated that BMI does not influence the clinical short-term outcomes following LNF, but long-term control of reflux by LNF in obese patients is worse than that in normal weight subjects. Therefore obesity is a relative contraindication to LNF.

## Figures and Tables

**Table 1:** Baseline characteristics of patients.

	All	Normal weight	Overweight	Obese	*p* value
*Patients number (%)*	201 (100)	43 (21.4)	89 (44.2)	69 (34.3)	
*AGE, y, mean (range)*	47.6 (28–86)	45.2 (31–84)	49.2 (34–86)	49.3 (28–77)	0.728
*Sex ratio number (%)*					
Female	118 (58.7)	28 (65.1)	51 (57.3)	39 (56.5)	0.752
Male	83 (41.3)	15 (34.9)	38 (42.7)	30 (43.4)	
*BMI, mean (range)*	27 (18.2–34.8)	21.6 (18.2–24.8)	28.3 (25.2–29.9)	32.1 (30.2–34.8)	
*Follow-up period, y, mean (range)*	16.5 (11–22)	16.1 (11–22)	15.8 (11–22)	15.6 (11–22)	

BMI = body mass index.

**Table 2:** Preoperative patient details.

	All	Normal weight	Overweight	Obese	*p* value
*PATIENTS number*	201	43	89	69	
*Symptom type number (%)*					
Typical	73 (36.3)	17 (39.5)	33 (37)	23 (33.3)	0.366
Mixed	123 (61.1)	25 (58.1)	54 (60.6)	44 (63.7)	
Atypical	5 (2.4)	1 (2.3)	2 (2.2)	2 (2.8)	
*Duration of symptoms, m, mean (range)*	24.4 (16–30)	24.5 (18–27)	24.9 (16–25)	26.8 (19–30)	0.286
*Endoscopic data number (%)*					
No esophagitis (NERD)	97 (48.2)	21 (48.8)	44 (49.4)	32 (46.3)	0.258
Esophagitis (ERD)	104 (51.7)	22 (51.1)	45 (50.5)	37 (53.6)	0.303
*Manometric data, mean (range):*					0.198
Total length of LES (cm)	2.6 (1–4.3)	2.8 (1.5–4.3)	2.4 (1–3.8)	2.3 (1.2–3.6)	
Abdominal length of LES (cm)	0.5 (0–1.8)	0.6 (1.1–1.5)	0.7 (0–1.8)	0.3 (0.6–1.5)	
Resting pressure of LES (mmHg)	7 (0–21)	6.3 (0–19.8)	8.2 (1.1–18.9)	6 (0.4–21)	
Amplitude of contractile waves (mmHg)	38 (5–142)	41 (7–141)	39 (8–142)	36 (7–139)	
*pH-metric data, mean (range)*					0.672
Percentage total time with pH < 4	18 (16–85)	19 (17–85)	16 (16–81)	17 (18–81)	
*Hiatal hernia number (%)*	29 (14.4)	3^*∗*^ (6.9)	13 (14.6)	13 (18.8)	0.001
Large (> 3 cm)					
no (%)	11 (37.9)	1	4	6	
Type 1	9	1	3	5	
Type 3	2	/	1	1	
Small (≤3 cm)					
Number (%)	18 (62.0)	2	9	7	
Type 1	15	2	8	5	
Type 3	3	/	1	2	
*Previous abdominal operation number (%)*	46 (22.8)	10 (23.2)	19 (21.3)	17 (24.6)	0.928

^*∗*^Significantly different versus other groups.

NERD (nonerosive reflux disease).

ERD (erosive reflux disease).

**Table 3:** Perioperative parameters.

	All	Normal weight	Overweight	Obese	*p* value
*Patients number*	201	43	89	69	
*Type of operation no (%)*					
Mini-Floppy Nissen	201 (100)	43 (100)	89 (100)	69 (100)	
*Duration of operation, min, mean (range)*	66.1 (24.1–120.2)	60.2 (24–72.8)	65.6 (33.4–68.9)	70.9 (51.2–120.2)^*∗*^	<0.0001
*Graft usage number (%)*	29 (14.4)	3 (6.9)^*∗*^	13 (14.6)	13 (18.8)	<0.0001
*Drains number (%)*	8 (3.9)	/^*∗*^	2 (2.2)	6 (8.6)	<0.0001
*Conversion rate number (%)*	13 (6.4)	2 (4.6)	6 (6.7)	5 (7.2)	0.952
*Postoperative*					
Hospitalization, days, mean (range)	2.5 (2–5)	2.1 (2-3)	2.3 (2–4)	2.6 (2–5)	0.612

^*∗*^Significantly different versus other groups.

**Table 4:** Long-term follow-up data.

	All	Normal weight	Overweight	Obese	*p* value
*Patients number*	201	43	89	69	
*Follow-up interval y, mean (range)*	16 (11–21)	15.8 (11–21)	15.5 (11–21)	15.1 (11–21)	
Incisional hernia	2	/	1	1	0.480
Dysphagia number (%)	34 (16.9)	8 (18.6)	15 (16.8)	11 (15.9)	0.150
<6 months	27 (13.4)	6 (13.9)	12 (13.4)	9 (13)	
>6 months	7 (3.4)	2 (4.6)	3 (3.3)	2 (2.8)	
Dilatation	4 (2)	1 (2.3)	2 (2.2)	1 (1.4)	0.082
Reoperation	3 (1.5)	1 (2.3)	1 (1.1)	1 (1.4)	0.088
Bloating number (%)	35 (17.4)	6 (13.9)	13 (14.6)	16 (23.1)	0.386
*Endoscopic data *number *(%)*					
No esophagitis (NERD)	33 (16.4)	4 (9.3)	11 (12.3)^*∗*^	18 (26.0)^*∗∗*^	0.0001
Esophagitis (ERD)	55 (27.3)	5 (11.6)	19 (21.3)^*∗*^	31 (44.9)^*∗∗*^	0.0001
Reflux recurrence number (%)	27 (13.4)	1 (2.3)	7 (7.8)^*∗*^	19 (27.5)^*∗∗*^	0.0001
Reoperation	7	/	1	6^*∗∗*^	0.0001
(A) Wrap undone	4	/	1	3	0.460
(B) Fundoplication herniation	3	/	/	3	0.322

^*∗*^ *p* < 0.001 versus normal weight.

^*∗∗*^ *p* < 0.0001 versus normal weight and *p* < 0.001 versus overweight.

NERD (nonerosive reflux disease).

ERD (erosive reflux disease).

**Table 5:** Reflux recurrence and esophageal 24-hour pH-impedance monitoring.

	All	Normal weight	Overweight	Obese
*Reflux recurrence*	27	1	7	19
*24-h esophageal pH recording *				
<5.8% acid exposure for total time^*∗*^	/	/	/	/
≥5.8% acid exposure for total time	27	1	7	19
<8.2% acid exposure in upright position^*∗*^	/	/	/	/
≥8.2% acid exposure in upright position	27	1	7	19
<3.5% acid exposure in supine position^*∗*^	/	/	/	/
≥3.5% acid exposure in supine position	27	1	7	19
*DeMeester Score*				
<14.7^*∗*^	/	/	/	/
≥14.7	27	1	7	19
*Symptom- reflux correlation number*				
SI ≥ 50% and SAP ≥ 95%	22	1	5	16
SI < 50% and SAP < 95%	5	/	2	3

^*∗*^No reflux recurrence.

SI = Symptom index.

SAP = Symptom association probability.
